# Recent advances in understanding autoimmune thyroid disease: the tallest tree in the forest of polyautoimmunity

**DOI:** 10.12688/f1000research.11535.1

**Published:** 2017-09-28

**Authors:** Sofie Bliddal, Claus Henrik Nielsen, Ulla Feldt-Rasmussen

**Affiliations:** 1 Department of Medical Endocrinology, Section 2132, Copenhagen University Hospital (Rigshospitalet), Blegdamsvej 9, 2100 Copenhagen, Denmark; 2 Institute for Inflammation Research, Center for Rheumatology and Spine Diseases, section 7521, Copenhagen University Hospital (Rigshospitalet), Blegdamsvej 9, 2100 Copenhagen, Denmark

**Keywords:** Thyroid autoimmunity, polyautoimmunity, immunology, autoimmune disease, Graves' disease, Hashimoto's thyroiditis, AITD, antibodies

## Abstract

Autoimmune thyroid disease (AITD) is often observed together with other autoimmune diseases. The coexistence of two or more autoimmune diseases in the same patient is referred to as polyautoimmunity, and AITD is the autoimmune disease most frequently involved. The occurrence of polyautoimmunity has led to the hypothesis that the affected patients suffer from a generalized dysregulation of their immune system. The present review summarizes recent discoveries unravelling the immunological mechanisms involved in autoimmunity, ranging from natural autoimmunity to disease-specific autoimmunity. Furthermore, the clinical grounds for considering AITD in a setting of polyautoimmunity are explored. A better understanding of these may pave the way for designing new treatment modalities targeting the underlying immune dysregulation when AITD appears in the context of polyautoimmunity.

## Introduction

Autoimmune thyroid diseases (AITDs), comprising the two main entities Hashimoto’s thyroiditis (HT) and Graves’ disease (GD), are the most common autoimmune diseases ^1^ and are often observed together with other autoimmune diseases. The occurrence of two or more diseases in the same patient is often referred to as polyautoimmunity ^2^. This has led to the hypothesis that many patients with autoimmune disease, in general, suffer from an underlying dysfunction of critical mechanisms ensuring self-tolerance. The present review examines recent advances in the understanding of immunological aspects involved in autoimmunity, ranging from physiological to disease-specific autoimmunity, and explores the clinical grounds for considering thyroid autoimmunity in a setting of polyautoimmunity.

## Immunological aspects of polyautoimmunity

### Natural polyautoimmunity

At the beginning of the 20th century, Paul Ehrlich demonstrated that animals do not produce antibodies against their own red blood cells and coined the term “horror autotoxicus” for the immune system’s reaction with the body’s own constituents ^3, 4^. At the same time, the normal occurrence of autoantibodies against spermatozoa was demonstrated ^5^. Since then, many studies have shown the existence of autoantibodies in healthy animals and humans, referred to as natural autoantibodies ^6, 7^. In general, natural autoantibodies show broad reactivity against more self- and non-self-antigens and are of the IgM isotype ^6, 8^, whereas disease-associated autoantibodies are of the IgG isotype and bind specific self-antigens with high affinity ^9, 10^. At least in mice, the subset of B cells producing natural autoantibodies appears to be positively selected for self-reactivity in the bone marrow ^11^. Although B cells and T cells binding to “self” with high affinity are negatively selected, self-reactive lymphocytes with low-affinity receptors are allowed to escape and enter the circulation but often without harmful consequences ^12, 13^. Thus, natural autoantibodies may serve a role in the clearance of aging cells and cells with tumor potential and may also exert anti-inflammatory control over B cells and T cells ^14, 15^. Natural autoimmunity should therefore should be distinguished from disease-associated autoimmunity.

### Loss of self-tolerance

**Figure 1.**
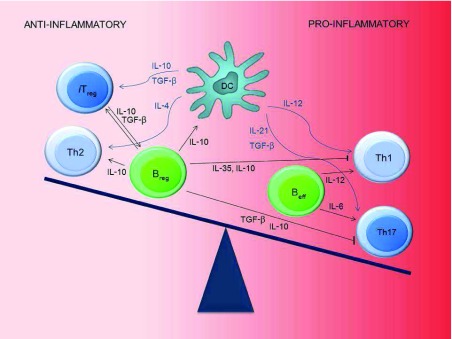
B-cell and T-cell subsets controlling autoimmunity. Peripheral self-tolerance is ensured by regulatory B cells and T cells. Autoimmunity (loss of self-tolerance) is considered to be due to a shift in favor of pro-inflammatory effector cells. B _eff_, effector B cells; B _reg_, regulatory B cells; DC, dendritic cell; IL, interleukin; iT _reg_, induced regulatory T cells; TGF-β, transforming growth factor beta; Th _1,2,17_, effector CD4 ^+^ T-cell subsets with different cytokine profiles.

Autoimmune disease is preceded by loss of immunological self-tolerance, which may occur at the central level (during the selection processes described above) or in the periphery. The adaptive immune system’s T cells and B cells usually keep the balance between reacting aptly toward foreign antigens and avoiding attack on “self”. Both T cells and B cells contain subsets of regulatory cells with an immunoregulatory cytokine profile on the one hand and effector cells that produce antibodies or secrete pro-inflammatory cytokines on the other ( Figure 1). Thus, the traditional view of a T helper type 1 (Th1)/Th2 dichotomy has been abandoned since it became clear that CD4 ^+^ effector T cells can differentiate into several subsets of Th cells which stimulate different parts of the immune system: for example, Th1 cells stimulate cellular immunity and the production of opsonizing antibodies, Th2 cells stimulate IgE production, and Th17 cells stimulate neutrophils. Th1 cells and Th17 cells have been shown to play detrimental roles in many autoimmune diseases, including HT and GD ^16, 17^. Furthermore, Th17 cells have been associated with chronic inflammation and autoimmune diseases such as systemic lupus erythematosus, Sjögren’s syndrome, and systemic sclerosis ^18^.

Regulatory T cells (Tregs) are primarily responsible for maintaining self-tolerance, and a protective role of Tregs has been demonstrated in several animal models of autoimmune diseases ^19, 20^. A subset of Tregs known as natural Tregs (nTregs) leave the thymus as fully differentiated cells, whereas another subset, inducible Tregs (iTregs), can be induced from naïve Th0 cells ^21^. Both subsets are characterized by the expression of the transcription factor forkhead box protein 3 (FOXP3) and mediate their action in part via the secretion of interleukin-10 (IL-10) and transforming growth factor beta (TGF-β) ^22^ and in part by mechanisms involving cell-to-cell contact ^23^. Loss of peripheral self-tolerance is considered to be the result of an overweight of Th17 cell response as compared with induced regulatory Th10 cell response ^18^. FOXP3 is involved in the peripheral self-tolerance processes by promoting the development of Tregs while inhibiting the differentiation of Th17 cells ^21^. Mutations of the *Foxp3* gene can cause the immune dysregulation, polyendocrinopathy, enteropathy, X-linked (IPEX) syndrome, an early-onset, life-threatening autoimmunity characterized by substantial polyautoimmunity and often death within two years of birth ^24^. Under less severe circumstances, overexpression of FOXP3Δ2, a splice variant of FOXP3 lacking exon 2 ^25^, may be associated with a shift toward a Th17 response, thereby increasing autoantibody production, as shown in a recent study of patients with HT ^26^. Accordingly, a skewed balance between Th17 cells and Tregs ^18, 21, 26^ as well as dysfunctional Tregs ^19^ have been demonstrated in autoimmune disease in humans. A shift between Th10 and Th17 cells may occur according to the cytokine milieu in the microenvironment ^18, 27^. Recently, regulatory B cells (Bregs) have received much attention as inhibitors of inflammatory and autoimmune responses by the production of regulatory cytokines (primarily IL-10) ^18^. Another factor that may contribute to the development of autoimmune disease is therefore a cytokine expression dominated by effector B cells rather than by Bregs. Thus, both Tregs and Bregs contribute to the maintenance of peripheral tolerance ( Figure 1).

### Development of autoimmune disease

**Figure 2.**
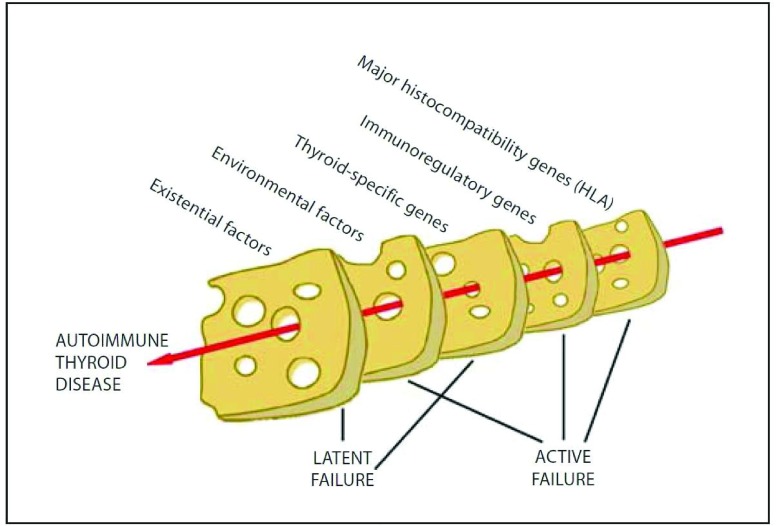
Etiology of thyroid autoimmunity. The development of autoimmune thyroid disease is a result of multiple events—a “Swiss cheese model”. Figure reproduced with kind permission from Weetman ^28^. HLA, human leukocyte antigen.

The transition from natural to clinically manifesting autoimmunity relies on an interplay between genetic predispositions and environmental events, as depicted in Weetman’s “Swiss cheese model” on AITD ( Figure 2) ^28^. Most autoimmune diseases are associated with specific variants of the human leukocyte antigen ( *HLA*) genes. Several other genetic polymorphisms have been associated with autoimmune diseases, particularly some located at the genes encoding cytotoxic T lymphocyte-associated 4 (CTLA-4) and the protein tyrosine phosphatase, non-receptor type 22 (PTPN22) ^29^. Each of these loci encodes molecules involved in the regulation of T cells. In particular, two rare syndromes illustrate the importance of T cells in maintaining self-tolerance: the first, autoimmune polyglandular syndrome type I (APS 1, also called APECED), is caused by defects in the autoimmune regulator ( *AIRE*) gene that mediates the induction of T-cell self-tolerance in the thymus ^30^. In patients with APS 1, multiple endocrine glands are dysfunctional. The second, FOXP3 deficiency, is associated with the IPEX syndrome, as mentioned above. Whereas APS 1 and IPEX syndrome are caused by mutation of a single gene, most cases of autoimmunity are likely the result of a broad range of genetic predispositions and environmental factors resulting in an imbalance of the peripheral self-tolerance mechanisms sustained by Tregs and Bregs ( Figure 1) ^18^. Thus, genetic polymorphisms in self-antigens, cytokines, estrogen receptors, and adhesion molecules have also been linked to the development of autoimmune disease ^28, 31– 36^, as have genes coding for apoptotic processes ^37^.

In addition to genetic variation, both post-transcriptional and post-translational events may contribute to the development of autoimmunity. Thus, translation of mRNA into protein may be regulated by binding of RNA-binding protein and microRNAs, and alternative splicing of mRNA may influence the functionality of encoded proteins ^38^. As mentioned above, certain splice variants of FOXP3 mRNA have been associated with autoimmune disease, including HT ^26, 39, 40^. An example of post-translational modification that enhances the immunogenicity of self-proteins is the conversion of arginine residues into citrulline residues, which has a major impact on autoantibody formation in rheumatoid arthritis ^41^.

Several environmental factors may trigger autoimmune disease in genetically predisposed individuals, including bacterial and viral infections, cigarette smoking, maternal-fetal microchimerism ^42, 43^, and exposure to chemical compounds (flame retardants and phthalates) ^44^, to mention but a few. Finally, being female predisposes to many autoimmune diseases, which may relate to hormonal factors and to X-chromosome inactivation patterns ^45, 46^.

### Autoimmune thyroid disease

AITD is increasingly viewed as a continuum not only including distinct disease entities—that is, HT, GD, subacute thyroiditis, primary myxedema, and Graves’ orbitopathy (GO)—but covering a spectrum of diseases affecting the thyroid gland. However, in most thyroid research and in clinical practice, the two main entities of AITD remain as GD and HT with typically opposing main clinical manifestations: hyperthyroidism and hypothyroidism, respectively. In line with a more flexible view of AITD, both HT and GD are heterogeneous and can cause both hyperthyroidism and hypothyroidism ^47^, even alternating between one form and the other ^48^. GD and HT are among the most common autoimmune diseases in Western countries ^49^. The pathogenesis is complex with a wide range of predisposing environmental, endogenous, and genetic factors, as described above and specified in relation to AITD in Box 1 ^45, 46, 50– 52^.

Box 1. **Risk factors for autoimmune thyroid disease**

Environmental

▪ Iodine consumption
▪ Smoking (possibly protective)
▪ Radiation
▪ Drugs (including biologic agents)
▪ Alcohol consumption (protective)
▪ Pollutants
▪ Selenium? Vitamin D?
▪ Infections ( *Yersinia enterocolitica*)?
▪ Improved hygiene?

Endogenous

▪ Female sex
▪ Parity
▪ Aging
▪ Stress hormones
▪ Fetal microchimerism

Genetic

▪ Chromosome abnormalities
▪ Human leukocyte antigen types?
▪ Single-nucleotide polymorphisms?

HT is characterized by a direct T-cell attack on the thyroid gland, leading to thyroiditis and subsequent exposure of thyroid antigens (thyroid peroxidase and thyroglobulin) against which antibodies are then produced. Thyroglobulin antibodies (TgAbs) and thyroid peroxidase antibodies (TPOAbs) are commonly associated with HT with a destructive pattern and are considered diagnostic for this disease. In any iodine-sufficient population, however, the prevalence of TPOAbs and TgAbs is much higher than that of clinical disease, amounting to approximately 15–25%, with the highest prevalence in females and increasing with age ^53^. GD is also primarily caused by a T-cell abnormality, but the hyperthyroidism associated with the disease is caused by the production of the pathognomonic thyrotropin receptor autoantibodies (TRAbs). The stimulating effect of TRAbs on thyrocytes probably has an influence on the formation of TgAbs and TPOAbs as well ^54^. GD (and less commonly HT) can be complicated by GO, an autoimmune reaction in the orbita causing fibroadipose tissue and extraocular muscles to increase in size with a risk of permanent damage to the optic nerve. Shared antigens between the thyroid and orbita have been suspected to be the target of the autoimmune response, and TRAbs are likely to play a part in the pathogenesis. A general inflammatory cytokine profile (mainly Th1-driven) and autoimmune reactions toward the insulin-like growth factor 1 receptor (IGF-1R) have been demonstrated as well ^55^.

### Polyautoimmune disease

Occasionally, patients suffer from more than one well-defined autoimmune disease. In the 1950s, it was recognized that certain “shared threads” characterized autoimmune diseases ^56, 57^, a notion which has since been the subject of much debate and research. Many terms have been suggested for such conditions: polyautoimmunity ^2, 58^, autoimmune diathesis ^57^, autoimmune tautology ^59^, or, in the case of three or more coexisting autoimmune diseases, multiple autoimmune syndromes ^60^. Lack of a common terminology challenges any comprehensive literature search and review. In the following, the term “polyautoimmunity” is used to describe the coexistence of two or more autoimmune diseases in the same patient.

Several endocrine syndromes including more than one autoimmune disease have been described (a sample of them is illustrated in Table 1 ^61– 64^). In polyglandular autoimmune syndrome type 2, Addison’s disease occurs with AITD or type 1 diabetes (as well as with other autoimmune presentations) ^61^. Such syndromes or classified clusters are rare in contrast to the presence of multiple autoantibodies (independent of clinical diagnoses) in patients with autoimmune disease. AITD, being the most common, is prevalent in most polyautoimmune syndromes and cases. The following sections describe the clinical findings related to thyroid autoimmunity in association with other autoimmunity.

**Table 1.** Polyautoimmune endocrine syndromes including thyroid disease.

Clusters	Proposed pathogenic mechanism
Autoimmune polyglandular syndromes ^61^	
Type I (autoimmune polyglandular syndrome type I or Whitaker syndrome): mucosal and cutaneous *Candida* infections, Addison’s disease, hyposplenism, hypoparathyroidism, and multiple autoimmune presentations (that is, hypothyroidism, hypogonadism, vitiligo, alopecia, pernicious anemia, and chronic autoimmune hepatitis)	Mutation of autoimmune regulator ( *AIRE*) gene involved in central tolerance development. Phenotype possibly affected by human leukocyte antigen (HLA) subtypes ^62^.
Type II (Schmidt’s syndrome): Addison’s disease and hypothyroidism or type 1A diabetes as well as pernicious anemia, primary hypogonadism, vitiligo, celiac disease, and myasthenia gravis (by some further classified in types III and IV according to specific entities above)	Polygenetic with increased risk of disease linked to specific HLA-DR and HLA-DQ genotypes ^63^
Immune dysregulation, polyendocrinopathy, enteropathy, X-linked (IPEX) syndrome ^24^ Immune dysfunction, enteropathy, dermatitis, autoimmune endocrinopathies (often type 1 diabetes and autoimmune thyroid disease), autoimmune skin diseases (that is, bullous pemphigoid), and multiple organ involvement	Mutation of *FOXP3* gene on the X chromosome
Multiple autoimmune syndromes ^60^	
Type I: myasthenia gravis, thymoma, polymyositis, and giant cell myocarditis	
Type II: Sjögren’s syndrome, rheumatoid arthritis, primary biliary cirrhosis, scleroderma, and autoimmune thyroid disorders	
Type III: autoimmune thyroid disease, myasthenia and/or thymoma, Sjögren’s syndrome, pernicious anemia, idiopathic thrombocytopenic purpura, Addison’s disease, insulin-dependent diabetes, vitiligo, autoimmune hemolytic anemia, and systemic lupus erythematosus	Genetic predisposition, with phenotype HLA B8 and/or DR3 or DR5 seeming to be an important factor
Thyrogastric cluster ^64^ Autoimmune thyroiditis, chronic gastritis/pernicious anemia, and autoimmune adrenalitis (Addison’s)	Polygenetic
Lupus-associated cluster ^64^ Autoimmune hemolytic anemia, immune thrombocytopenia, systemic lupus erythematosus, rheumatoid arthritis, autoimmune hepatitis, and Sjögren’s syndrome	Polygenetic
Trisomy 21 and Turner syndrome ^64^ Chronic thyroiditis, type 1A diabetes, and others	Chromosomal abnormalities
Kearns-Sayre syndrome ^64^ External ophthalmoplegia, retinal degeneration, diabetes, thyroiditis, and hypoparathyroidism	Mitochondrial myopathy

## Thyroid autoimmunity in association with polyautoimmunity

### Thyroid autoimmunity in other autoimmune disease

It is beyond the scope of this review to cover the numerous case reports describing polyautoimmunity. Instead, the present review will focus on clinical studies on polyautoimmunity involving AITD. In many studies, no distinction has been made between the various clinical phenotypes of AITD, and TgAbs, TPOAbs, and even TRAbs are often just referred to as “thyroid autoantibodies”, making reported percentages indicative only.

In a Colombian study ^2^, patterns of clustering were investigated among 1,083 patients. The patients had systemic lupus erythematosus, rheumatoid arthritis, systemic sclerosis, or multiple sclerosis. AITD was the most prevalent disease, coexisting with systemic sclerosis in 23% of cases, rheumatoid arthritis in 21% of cases, systemic lupus erythematosus in 18% of cases, and multiple sclerosis in 9% ^2^. No control group was provided to substantiate the findings. Among 479 patients with primary Sjögren’s syndrome, Zeher *et al*. found 21% with AITD ^65^. Another study from the same group showed that HT was significantly more prevalent than GD in patients with mixed connective tissue disease, rheumatoid arthritis, and Sjögren’s syndrome and that both AITDs were much more prevalent in such patients than in the background population ^66^. A total of 8.2% of investigated patients with the investigated systemic autoimmune diseases had AITD based on clinical evaluation, imaging, and fine needle aspiration cytology ^66^.

A few studies of autoantibody prevalence have included healthy controls. Nakamura *et al*. ^67^ found significant increases in TgAb and TPOAb positivity in all investigated autoimmune diseases compared with healthy controls: up to 50% in type 1 diabetes, 55% in autoimmune liver disease, 26% in myasthenia gravis, and 34% in connective tissue diseases ^67^. Only patients with type 1 diabetes had TRAbs more often than healthy controls (20% versus 0%, *P* <0.01). Interestingly, Liao *et al*. ^68^ found no significant difference in thyroid autoantibody positivity between 1,290 patients with rheumatoid arthritis and 1,236 controls without rheumatic disease (TPOAbs 15.6% versus 15.9%, TgAbs 1.1% versus 0.7%, *P* >0.05) ^68^. We have previously found TPOAbs in 5% of patients with rheumatoid arthritis, 19% with type 1 diabetes mellitus, 11% with Sjögren’s syndrome, 56% with pernicious anemia, and 22% with primary biliary cirrhosis compared with 98% with HT and 7% in healthy controls, which is in keeping with the above-mentioned increased risk of autoimmune disease and not only positivity for autoantibody ^69^.

### Non-thyroid autoantibodies and other autoimmune disease in autoimmune thyroid disease

Few studies have investigated the prevalence of non-thyroid autoantibodies or other autoimmune diseases in patients with AITD. In a recently published prospective study, 3,069 patients with chronic AITD were compared with 1,023 patients with non-toxic nodular goiter and 1,023 healthy controls ^70^. Diagnosis of autoimmune disease was confirmed by a specialist according to scientific societies’ criteria. Several of the concurrent autoimmune diseases were significantly more prevalent in patients with AITD than in the healthy controls. However, only three concurrent diseases had a prevalence of more than 2% in the AITD group: chronic autoimmune gastritis, vitiligo, and rheumatoid arthritis. Among observed clusters of disease, AITD together with chronic autoimmune gastritis and vitiligo formed a cluster with a significantly increased prevalence in the group of patients with AITD (12 patients versus 0 in other groups, *P* = 0.02) ^70^.

In a Japanese population of patients with AITD, anti-glutamic acid decarboxylase antibodies were the most prevalent non-thyroid autoantibodies, being present in 6.4% of GD and in 4.6% of patients with HT ^67^, thus confirming historical links between insulin antibodies and thyroid autoimmunity ^71^. In a study of more than 3,000 UK patients with AITD, there was a significantly increased relative risk of any other autoimmune disease screened for compared with the background population ^72^. Thus, 14.3% of the 495 patients with HT and 9.7% with GD had at least one other autoimmune disease, mainly type 1 diabetes, rheumatoid arthritis, and pernicious anemia. Wiebolt *et al*. investigated 359 patients with HT and 523 with GD and found differing patterns of autoantibody clustering in the two diseases ^73^. Thus, adrenal autoimmunity combined with beta-cell or gastric autoimmunity was more common in patients with HT compared with patients with GD ^73^.

### Epidemiological studies

Lack of power in clinical studies is made up for in epidemiologic studies. Eaton *et al*. included data from more than 5 million Danes from hospital registries to investigate the prevalence of 31 specified autoimmune diseases (based on the International Classification of Diseases 10th Revision (ICD10) classification system) ^74, 75^. Although data were limited to that of specialized care, the estimated lifetime prevalence of any autoimmune disease was 5.3%, and AITD most often coexisted with adrenal disease (odds ratio (OR) 12.9), alopecia areata (OR 11.4), vitiligo (OR 7.9), and pernicious anemia (OR 5.6) ^75^. However, compared with connective tissue diseases in particular, AITD showed limited overlap with other autoimmune diseases. As many patients with autoimmune disease are handled in general practice, a British study used the United Kingdom General Practice Research Database to study the intra-individual risk of polyautoimmunity ^76^. Along with large numbers of patients with insulin-dependent diabetes mellitus, rheumatoid arthritis, and multiple sclerosis, 26,198 patients with AITD were included. Patients with either AITD or rheumatoid arthritis were at risk of developing the other disease (sex-specific standardized incidence rate of 130.4–162.0) ^76^. The risk of patients with type 1 diabetes having AITD was increased significantly in comparison with the background population having AITD; with a prevalence six times higher in males and a prevalence four times higher in females. This is in keeping with at least one previous cohort study, where patients with two autoimmune diseases, and thus probably stronger influence of genetic than environmental or other inherent factors, seemed to show a diminished or even abolished female preponderance of autoimmune diseases that is otherwise observed in the general population ^77^. Thus, the *a priori* likelihood of contracting a second autoimmune disease is higher in males than in females with one autoimmune disease, which should be taken into account when managing male patients with AITD. Despite many inherent limitations, epidemiological studies provide further evidence of an increased risk of polyautoimmunity in patients with AITD. Without proving causality, the association between AITD and other autoimmune diseases seems well substantiated and with no strict distinction between organ-specific and non-organ-specific autoimmune disease.

## Clinical consequences of polyautoimmunity in thyroid autoimmunity

### Clinical impact of (poly)autoimmunity

The chronic nature of autoimmune disease implies high socioeconomic costs as well as a profound impact on the patient’s health and well-being ^78^. The occurrence of polyautoimmunity rather than an isolated autoimmune disease may greatly affect prognosis. In a Dutch study, TPOAb positivity in patients with rheumatoid arthritis was predictive of disease activity—measured by the Disease Activity Score using 28 joint counts (DAS28)—and of increased carotid intima-media thickness at a 2-year follow-up ^79^. The latter finding may indicate an increased risk of cardiovascular disease in patients with polyautoimmunity in accordance with the well-established association between increased inflammation and risk of cardiovascular disease ^80, 81^. In studies of reproductive failure, especially autoantibodies involved in the anti-phospholipid syndrome (that is, anti-cardiolipin antibodies) ^82^ but also thyroid autoantibodies ^83, 84^ have been associated with worsened pregnancy outcomes. In a study by Iijima *et al*. ^85^, several autoantibodies were analyzed, and women with autoantibodies against one or more self-antigens had a significantly increased risk of miscarriage. However, only 5% of all women (25.8% of autoantibody-positive women) had multiple autoantibodies ^85^. In women with recurrent pregnancy loss, a thorough examination of possible polyautoimmune etiology is therefore warranted, and immunotherapy appears to be beneficial ^86^.

Few studies indicate an impact of an underlying immunological tolerance breach rather than thyroid dysfunction on the symptomatology involved in AITD. Abnormal brain perfusion in patients with euthyroid HT compared with controls has led to a hypothesis of an autoimmune component in decreased vascularization, possibly causing psychiatric symptoms in patients with AITD ^87, 88^. Also, a study of patient-reported outcomes (PROs) in Danish patients with AITD found a significant continuous reduction of outcomes, including physical functioning despite euthyroidism ^89^. A subsequent study found reduced PROs in patients with GD compared with patients with toxic nodular goiter but failed to show an association with thyroid antibody levels ^90^. Conversely, in studies of patients with systemic sclerosis and myasthenia gravis, those with coexistent AITD were less affected by their disease than patients without thyroid autoimmunity ^91, 92^. Thus, quantitative increments of autoimmune diagnoses are not always associated with a worsened prognosis.

The possible negative attribution of underlying autoimmunity (for example, GD) rather than thyroid function impairment (for example, multinodular goiter) needs further investigation. The presence of a more general but undiagnosed polyautoimmunity could explain the poorer outcomes of treatment. This has in recent years been acknowledged by endocrinologists and is now introduced in clinical guidelines (for example, for the treatment of hypothyroidism) ^93^. Before combination therapy with thyroxine and triiodothyronine is considered, a search for other autoimmune diseases in a patient with AITD and poor PROs is now mandatory ^93^. Thus, it has become relevant to both the clinician and the patient with AITD to be aware of the possibility of polyautoimmunity, not only as an explanatory model of prolonged symptoms but also with respect to a possible altered prognosis of disease and treatment outcome.

### The role of immunomodulatory biologic drugs

The prevalence of thyroid and other autoimmunity will likely be affected by the increasing administration of biologic agents in cancer treatment and internal medicine, developed to target specific parts of the immune system. In multiple sclerosis patients treated with the T cell–depleting anti-CD52 monoclonal antibody alemtuzumab, one third of patients developed GD between 6 and 31 weeks after treatment ^94^. This was likely due to loss of T cell–mediated immunoregulation. Also, in patients with chronic hepatitis treated with interferon-alpha, up to 40% developed thyroid autoantibodies and 15% developed clinical AITD ^95, 96^. Stefan *et al*. found a direct impact of interferon-alpha on the activity of a thyroglobulin promotor region with a specific single-nucleotide polymorphism involved in AITD ^97^. Finally, biologic drugs stimulating the patient’s own immune response against cancer cells have proven efficient in fighting cancers but may induce various autoimmune diseases. Among such drugs are the checkpoint inhibitors ipilimumab (a monoclonal antibody targeting the CTLA-4 receptor) and nivolumab and pembrolizumab (blocking “programmed cell death 1” proteins on T cells, thus promoting anti-tumor activity). Autoimmune hypophysitis is seen in 4% of patients treated with ipilimumab and in 1% of patients treated with nivolumab or pembrolizumab. Combined treatment with ipilimumab and nivolumab increases the hypophysitis rate to 8% ^98^. In these patients with autoimmune hypophysitis, secondary adrenal insufficiency develops in up to 100%, hypogonadotropic hypogonadism in up to 85%, and central hypothyroidism in up to 100% ^98^. More rarely, autoimmune primary thyroid affection or adrenal insufficiency occurs ^99^. Thus, the use of such drugs should entail a standardized monitoring program to check for endocrinopathies (i.e. life-threatening adrenal insufficiency) and thorough patient information including which symptoms to react upon.

On the other hand, biologic agents may offer new options for treating (poly)autoimmune diseases, including otherwise treatment-resistant AITD. Treatment of rheumatic arthritis patients with biologic agents seems to be safe and to possibly have a positive effect on concurrent AITD ^100^. In a small study, the treatment of 138 patients with rheumatoid arthritis with the tumor necrosis factor-alpha inhibitor adalimumab led to concurrent improvement of hypothyroidism as well as a reduction in TPOAb levels ^101^. Incorporation of rituximab, a B cell–depleting agent, has shown promising results in patients with GO ^102– 104^ in addition to ameliorating numerous other autoimmune diseases, such as rheumatoid arthritis ^103, 105^, multiple sclerosis ^106^, and type 1 diabetes ^107^. In a recent study, inhibition of the IGF-1R by use of teprotumumab, an antibody targeting the IGF-1R, also significantly improved outcome in 88 patients with GO ^108^. The field of drugs targeting immune-mediated diseases is quickly evolving, and numerous drugs have been developed and are undergoing clinical trials ^109^. Although the mechanisms triggering loss of self-tolerance are still unclear, the increasing knowledge about B-cell and T-cell subsets and different cytokine milieus provides interesting perspectives for refined treatment options in (poly)autoimmune diseases including treatment-resistant AITD. For a summary of learning points, see Box 2.

Box 2. **Learning points**

- Polyautoimmunity in patients with autoimmune thyroid disease is common but not part of routine screening.
- Polyautoimmunity may increase morbidity due to inflammation.
- Patients with continuous complaints despite euthyroidism may benefit from wider immunological assessment.
- Interdisciplinary assessment is beneficial for polyautoimmune patients.
- Immunomodulatory biologic drugs may be a future treatment option in thyroid autoimmune patients with multiple associated autoantibodies.

## Conclusions

As illustrated above, AITD is often represented in patients with polyautoimmunity, and patients with AITD have an increased risk of developing other autoimmune diseases. In AITD patients with remaining complaints despite euthyroidism or patients presenting with new symptoms, polyautoimmunity should come to mind. Furthermore, the concept of polyautoimmunity may call for a rethinking of treatment strategies in patients with AITD. Current treatment of AITD has focused on securing thyroid hormone homeostasis, but in cases with polyautoimmunity more attention should be drawn to the identification and treatment of the underlying immune dysregulation and inflammation. In years to come, immunomodulatory biologic drugs may serve this purpose.

## References

[1] Weetman A DeGroot LJ: Autoimmunity to the Thyroid Gland.In: De Groot LJ, Chrousos G, Dungan K, Feingold KR, Grossman A, Hershman JM, *et al.*, editors. Endotext. South Dartmouth (MA);2000. 25905407

[2] Rojas-Villarraga A Amaya-Amaya J Rodriguez-Rodriguez A: Introducing polyautoimmunity: secondary autoimmune diseases no longer exist. *Autoimmune Dis.* 2012;2012: 254319. 10.1155/2012/254319 22454759 PMC3290803

[3] Ehrlich P: Verh Ges Dtsch Naturforsch.1902;1:250–275.

[4] Welch WH: The Huxley Lecture on Recent Studies of Immunity, with Special Reference to their Bearing on Pathology: Delivered at the Opening of the Winter Session of Charing Cross Hospital Medical School on October 1st, 1902. *Br Med J.* 1902;2(2180):1105–14. 20760475 10.1136/bmj.2.2180.1105 PMC2401880

[5] Metalnikoff S: Ann Inst Pasteur.1900;14:577–589.

[6] Avrameas S Selmi C: Natural autoantibodies in the physiology and pathophysiology of the immune system. *J Autoimmun.* 2013;41:46–9. 10.1016/j.jaut.2013.01.006 23384670

[7] Nakamura M Burastero SE Ueki Y: Probing the normal and autoimmune B cell repertoire with Epstein-Barr virus. Frequency of B cells producing monoreactive high affinity autoantibodies in patients with Hashimoto’s disease and systemic lupus erythematosus. *J Immunol.* 1988;141(12):4165–72. 2848890

[8] Berneman A Guilbert B Eschrich S: IgG auto- and polyreactivities of normal human sera. *Mol Immunol.* 1993;30(16):1499–510. 8232336 10.1016/0161-5890(93)90458-n

[9] Nielsen CH Bendtzen K: Immunoregulation by naturally occurring and disease-associated autoantibodies: binding to cytokines and their role in regulation of T-cell responses. *Adv Exp Med Biol.* 2012;750:116–32. 10.1007/978-1-4614-3461-0_9 22903670 PMC7123141

[10] Nielsen CH Brix TH Gardas A: Epitope recognition patterns of thyroid peroxidase autoantibodies in healthy individuals and patients with Hashimoto’s thyroiditis*. *Clin Endocrinol (Oxf).* 2008;69(4):664–8. 10.1111/j.1365-2265.2008.03245.x 18363888

[11] Hayakawa K Asano M Shinton SA: Positive selection of natural autoreactive B cells. *Science.* 1999;285(5424):113–6. 10.1126/science.285.5424.113 10390361

[12] Nossal GJ: Negative selection of lymphocytes. *Cell.* 1994;76(2):229–39. 10.1016/0092-8674(94)90331-X 8293461

[13] Webb S Morris C Sprent J: Extrathymic tolerance of mature T cells: clonal elimination as a consequence of immunity. *Cell.* 1990;63(6):1249–56. 10.1016/0092-8674(90)90420-J 2148123

[14] Lutz HU Binder CJ Kaveri S: Naturally occurring auto-antibodies in homeostasis and disease. *Trends Immunol.* 2009;30(1):43–51. 10.1016/j.it.2008.10.002 19058756

[15] Hurez V Dietrich G Kaveri SV: Polyreactivity is a property of natural and disease-associated human autoantibodies. *Scand J Immunol.* 1993;38(2):190–6. 10.1111/j.1365-3083.1993.tb01712.x 8346418

[16] Rapoport B McLachlan SM: Graves’ hyperthyroidism is antibody-mediated but is predominantly a Th1-type cytokine disease. *J Clin Endocrinol Metab.* 2014;99(11):4060–1. 10.1210/jc.2014-3011 25210884 PMC4223433

[17] Figueroa-Vega N Alfonso-Pérez M Benedicto I: Increased circulating pro-inflammatory cytokines and Th17 lymphocytes in Hashimoto’s thyroiditis. *J Clin Endocrinol Metab.* 2010;95(2):953–62. 10.1210/jc.2009-1719 20016049

[18] Papp G Boros P Nakken B: Regulatory immune cells and functions in autoimmunity and transplantation immunology. *Autoimmun Rev.* 2017;16(5):435–44. 10.1016/j.autrev.2017.03.011 28286106

[19] Ramos-Leví AM Marazuela M: Pathogenesis of thyroid autoimmune disease: the role of cellular mechanisms. *Endocrinol Nutr.* 2016;63(8):421–9. 10.1016/j.endonu.2016.04.003 27234136

[20] Morris GP Brown NK Kong YM: Naturally-existing CD4(+)CD25(+)Foxp3(+) regulatory T cells are required for tolerance to experimental autoimmune thyroiditis induced by either exogenous or endogenous autoantigen. *J Autoimmun.* 2009;33(1):68–76. 10.1016/j.jaut.2009.03.010 19375891 PMC2706097

[21] González-Amaro R Marazuela M: T regulatory (Treg) and T helper 17 (Th17) lymphocytes in thyroid autoimmunity. *Endocrine.* 2016;52(1):30–8. 10.1007/s12020-015-0759-7 26475497

[22] Roncarolo MG Gregori S Battaglia M: Interleukin-10-secreting type 1 regulatory T cells in rodents and humans. *Immunol Rev.* 2006;212:28–50. 10.1111/j.0105-2896.2006.00420.x 16903904

[23] von Boehmer H: Mechanisms of suppression by suppressor T cells. *Nat Immunol.* 2005;6(4):338–44. 10.1038/ni1180 15785759

[24] Bennett CL Christie J Ramsdell F: The immune dysregulation, polyendocrinopathy, enteropathy, X-linked syndrome (IPEX) is caused by mutations of *FOXP3*. *Nat Genet.* 2001;27(1):20–1. 10.1038/83713 11137993

[25] Allan SE Passerini L Bacchetta R: The role of 2 FOXP3 isoforms in the generation of human CD4+ Tregs. *J Clin Invest.* 2005;115(11):3276–84. 10.1172/JCI24685 16211090 PMC1242190

[26] Kristensen B Hegedüs L Madsen HO: Altered balance between self-reactive T helper (Th)17 cells and Th10 cells and between full-length forkhead box protein 3 (FoxP3) and FoxP3 splice variants in Hashimoto’s thyroiditis. *Clin Exp Immunol.* 2015;180(1):58–69. 10.1111/cei.12557 25412700 PMC4367094

[27] Yanaba K Bouaziz J Haas KM: A regulatory B cell subset with a unique CD1dhiCD5+ phenotype controls T cell-dependent inflammatory responses. *Immunity.* 2008;28(5):639–50. 10.1016/j.immuni.2008.03.017 18482568

[28] Weetman AP: The immunopathogenesis of chronic autoimmune thyroiditis one century after hashimoto. *Eur Thyroid J.* 2013;1(4):243–50. 10.1159/000343834 24783026 PMC3821488

[29] Brand O Gough S Heward J: *HLA, CTLA-4 and PTPN22*: The shared genetic master-key to autoimmunity? *Expert Rev Mol Med.* 2005;7(23):1–15. 10.1017/S1462399405009981 16229750

[30] Mathis D Benoist C: Aire. *Annu Rev Immunol.* 2009;27:287–312. 10.1146/annurev.immunol.25.022106.141532 19302042

[31] Caputo M Rivolta CM Mories T: Analysis of thyroglobulin gene polymorphisms in patients with autoimmune thyroiditis. *Endocrine.* 2010;37(3):389–95. 10.1007/s12020-010-9317-5 20960158

[32] Zenewicz LA Abraham C Flavell RA: Unraveling the genetics of autoimmunity. *Cell.* 2010;140(6):791–7. 10.1016/j.cell.2010.03.003 20303870 PMC3491807

[33] Johar AS Mastronardi C Rojas-Villarraga A: Novel and rare functional genomic variants in multiple autoimmune syndrome and Sjögren’s syndrome. *J Transl Med.* 2015;13:173. 10.1186/s12967-015-0525-x 26031516 PMC4450850

[34] Johar AS Anaya JM Andrews D: Candidate gene discovery in autoimmunity by using extreme phenotypes, next generation sequencing and whole exome capture. *Autoimmun Rev.* 2015;14(3):204–9. 10.1016/j.autrev.2014.10.021 25447288

[35] Chu X Pan CM Zhao SX: A genome-wide association study identifies two new risk loci for Graves’ disease. *Nat Genet.* 2011;43(9):897–901. 10.1038/ng.898 21841780

[36] Lee HS Kang J Yang S: Susceptibility influence of a *PTPN22* haplotype with thyroid autoimmunity in Koreans. *Diabetes Metab Res Rev.* 2011;27(8):878–82. 10.1002/dmrr.1265 22069277

[37] Johar A Sarmiento-Monroy JC Rojas-Villarraga A: Definition of mutations in polyautoimmunity. *J Autoimmun.* 2016;72:65–72. 10.1016/j.jaut.2016.05.003 27209085

[38] Chen JQ Papp G Szodoray P: The role of microRNAs in the pathogenesis of autoimmune diseases. *Autoimmun Rev.* 2016;15(12):1171–80. 10.1016/j.autrev.2016.09.003 27639156

[39] Link M Salur L Kisand K: Higher FoxP3 mRNA expression in peripheral blood mononuclear cells of GAD65 or IA-2 autoantibody-positive compared with autoantibody-negative persons. *APMIS.* 2008;116(10):896–902. 10.1111/j.1600-0463.2008.00889.x 19132983

[40] Ryder LR Bartels EM Woetmann A: FoxP3 mRNA splice forms in synovial CD4+ T cells in rheumatoid arthritis and psoriatic arthritis. *APMIS.* 2012;120(5):387–96. 10.1111/j.1600-0463.2011.02848.x 22515293

[41] Catrina AI Svensson CI Malmström V: Mechanisms leading from systemic autoimmunity to joint-specific disease in rheumatoid arthritis. *Nat Rev Rheumatol.* 2017;13(2):79–86. 10.1038/nrrheum.2016.200 27974851

[42] Lepez T Vandewoestyne M Deforce D: Fetal microchimeric cells in autoimmune thyroid diseases: harmful, beneficial or innocent for the thyroid gland? *Chimerism.* 2013;4(4):111–8. 10.4161/chim.25055 23723083 PMC3921191

[43] Stevens AM: Maternal microchimerism in health and disease. *Best Pract Res Clin Obstet Gynaecol.* 2016;31:121–30. 10.1016/j.bpobgyn.2015.08.005 26612343

[44] Mynster Kronborg T Frohnert Hansen J Nielsen CH: Effects of the Commercial Flame Retardant Mixture DE-71 on Cytokine Production by Human Immune Cells. *PLoS One.* 2016;11(4):e0154621. 10.1371/journal.pone.0154621 27128973 PMC4851365

[45] Gleicher N Barad DH: Gender as risk factor for autoimmune diseases. *J Autoimmun.* 2007;28(1):1–6. 10.1016/j.jaut.2006.12.004 17261360

[46] Brix TH Hegedüs L: Twin studies as a model for exploring the aetiology of autoimmune thyroid disease. *Clin Endocrinol (Oxf).* 2012;76(4):457–64. 10.1111/j.1365-2265.2011.04318.x 22168537

[47] Bech K Bliddal H Feldt-Rasmussen U: Heterogeneity of autoimmune thyroiditis. *Allergy.* 1984;39(4):239–47. 10.1111/j.1398-9995.1984.tb00861.x 6375440

[48] DeGroot LJ: Diagnosis and Treatment of Graves’ Disease.In: De Groot LJ, Chrousos G, Dungan K, Feingold KR, Grossman A, Hershman JM, *et al.*, editors. Endotext. South Dartmouth (MA);2000. 25905403

[49] Vanderpump MP: The epidemiology of thyroid disease. *Br Med Bull.* 2011;99:39–51. 10.1093/bmb/ldr030 21893493

[50] Pedersen IB Knudsen N Carlé A: A cautious iodization programme bringing iodine intake to a low recommended level is associated with an increase in the prevalence of thyroid autoantibodies in the population. *Clin Endocrinol (Oxf).* 2011;75(1):120–6. 10.1111/j.1365-2265.2011.04008.x 21521277

[51] Jørgensen KT Pedersen BV Nielsen NM: Childbirths and risk of female predominant and other autoimmune diseases in a population-based Danish cohort. *J Autoimmun.* 2012;38(2–3):J81–7. 10.1016/j.jaut.2011.06.004 21813263

[52] Hansen PS Brix TH Iachine I: The relative importance of genetic and environmental effects for the early stages of thyroid autoimmunity: a study of healthy Danish twins. *Eur J Endocrinol.* 2006;154(1):29–38. 10.1530/eje.1.02060 16381988

[53] Pedersen IB Knudsen N Jørgensen T: Thyroid peroxidase and thyroglobulin autoantibodies in a large survey of populations with mild and moderate iodine deficiency. *Clin Endocrinol (Oxf).* 2003;58(1):36–42. 10.1046/j.1365-2265.2003.01633.x 12519410

[54] Rasmussen AK Feldt-Rasmussen U Brandt M: Thyrotropin stimulates specifically the expression of the autoantibody binding domains of the thyroperoxidase molecule. *Autoimmunity.* 1999;29(4):323–31. 10.3109/08916939908994752 10433088

[55] Smith TJ Hegedüs L: Graves' Disease. *N Engl J Med.* 2016;375:1552–65. 10.1056/NEJMra1510030 27797318

[56] WITEBSKY E ROSE NR TERPLAN K: Chronic thyroiditis and autoimmunization. *J Am Med Assoc.* 1957;164(13):1439–47. 10.1001/jama.1957.02980130015004 13448890

[57] Rose NR: Autoimmune diseases: tracing the shared threads. *Hosp Pract (1995).* 1997;32(4):147–54. 10.1080/21548331.1997.11443469 9109812

[58] Sheehan NJ Stanton-King K: Polyautoimmunity in a young woman. *Br J Rheumatol.* 1993;32(3):254–6. 10.1093/rheumatology/32.3.254 8448621

[59] Anaya JM Castiblanco J Rojas-Villarraga A: The multiple autoimmune syndromes. A clue for the autoimmune tautology. *Clin Rev Allergy Immunol.* 2012;43(3):256–64. 10.1007/s12016-012-8317-z 22648455

[60] Humbert P Dupond JL: [Multiple autoimmune syndromes]. *Ann Med Interne (Paris).* 1988;139(3):159–68. 3059902

[61] Eisenbarth GS Gottlieb PA: Autoimmune polyendocrine syndromes. *N Engl J Med.* 2004;350(20):2068–79. 10.1056/NEJMra030158 15141045

[62] Halonen M Eskelin P Myhre AG: AIRE mutations and human leukocyte antigen genotypes as determinants of the autoimmune polyendocrinopathy-candidiasis-ectodermal dystrophy phenotype. *J Clin Endocrinol Metab.* 2002;87(6):2568–74. 10.1210/jcem.87.6.8564 12050215

[63] Myhre AG Undlien DE Løvås K: Autoimmune adrenocortical failure in Norway autoantibodies and human leukocyte antigen class II associations related to clinical features. *J Clin Endocrinol Metab.* 2002;87(2):618–23. 10.1210/jcem.87.2.8192 11836294

[64] Mackay IR: Clustering and commonalities among autoimmune diseases. *J Autoimmun.* 2009;33(3–4):170–7. 10.1016/j.jaut.2009.09.006 19837564

[65] Zeher M Horvath IF Szanto A: Autoimmune thyroid diseases in a large group of Hungarian patients with primary Sjögren's syndrome. *Thyroid.* 2009;19(1):39–45. 10.1089/thy.2007.0398 19119981

[66] Biró E Szekanecz Z Czirják L: Association of systemic and thyroid autoimmune diseases. *Clin Rheumatol.* 2006;25(2):240–5. 10.1007/s10067-005-1165-y 16247581

[67] Nakamura H Usa T Motomura M: Prevalence of interrelated autoantibodies in thyroid diseases and autoimmune disorders. *J Endocrinol Invest.* 2008;31(10):861–5. 10.1007/BF03346432 19092289

[68] Liao KP Kurreeman F Li G: Associations of autoantibodies, autoimmune risk alleles, and clinical diagnoses from the electronic medical records in rheumatoid arthritis cases and non-rheumatoid arthritis controls. *Arthritis Rheum.* 2013;65(3):571–81. 10.1002/art.37801 23233247 PMC3582761

[69] Feldt-Rasmussen U Høier-Madsen M Bech K: Anti-thyroid peroxidase antibodies in thyroid disorders and non-thyroid autoimmune diseases. *Autoimmunity.* 1991;9(3):245–54. 10.3109/08916939109007650 1777557

[70] Fallahi P Ferrari SM Ruffilli I: The association of other autoimmune diseases in patients with autoimmune thyroiditis: Review of the literature and report of a large series of patients. *Autoimmun Rev.* 2016;15(12):1125–8. 10.1016/j.autrev.2016.09.009 27639841

[71] Di Mario U Perfetti R Anastasi E: Autoantibodies to insulin do appear in non-diabetic patients with autoimmune disorders: comparison with anti-immunoglobulin antibodies and other autoimmune phenomena. *Acta Endocrinol (Copenh).* 1990;122(3):303–8. 10.1530/acta.0.1220303 2183533

[72] Boelaert K Newby PR Simmonds MJ: Prevalence and relative risk of other autoimmune diseases in subjects with autoimmune thyroid disease. *Am J Med.* 2010;123(2):183.e1–9. 10.1016/j.amjmed.2009.06.030 20103030

[73] Wiebolt J Achterbergh R den Boer A: Clustering of additional autoimmunity behaves differently in Hashimoto's patients compared with Graves' patients. *Eur J Endocrinol.* 2011;164(5):789–94. 10.1530/EJE-10-1172 21378091

[74] Eaton WW Pedersen MG Atladóttir HO: The prevalence of 30 ICD-10 autoimmune diseases in Denmark. *Immunol Res.* 2010;47(1–3):228–31. 10.1007/s12026-009-8153-2 20066507 PMC2892249

[75] Eaton WW Rose NR Kalaydjian A: Epidemiology of autoimmune diseases in Denmark. *J Autoimmun.* 2007;29(1):1–9. 10.1016/j.jaut.2007.05.002 17582741 PMC2717015

[76] Somers EC Thomas SL Smeeth L: Are individuals with an autoimmune disease at higher risk of a second autoimmune disorder? *Am J Epidemiol.* 2009;169(6):749–55. 10.1093/aje/kwn408 19224981

[77] Ottesen M Feldt-Rasmussen U Andersen J: Thyroid function and autoimmunity in pernicious anemia before and during cyanocobalamin treatment. *J Endocrinol Invest.* 1995;18(2):91–7. 10.1007/BF03349707 7629393

[78] Anaya JM: The diagnosis and clinical significance of polyautoimmunity. *Autoimmun Rev.* 2014;13(4–5):423–6. 10.1016/j.autrev.2014.01.049 24424171

[79] Raterman HG Voskuyl AE Simsek S: Increased progression of carotid intima media thickness in thyroid peroxidase antibodies-positive rheumatoid arthritis patients. *Eur J Endocrinol.* 2013;169(6):751–7. 10.1530/EJE-13-0394 24005313

[80] Willeit P Thompson SG Agewall S: Inflammatory markers and extent and progression of early atherosclerosis: Meta-analysis of individual-participant-data from 20 prospective studies of the PROG-IMT collaboration. *Eur J Prev Cardiol.* 2016;23(2):194–205. 10.1177/2047487314560664 25416041 PMC4544641

[81] Caforio AL Wagner R Gill JS: Organ-specific cardiac antibodies: serological markers for systemic hypertension in autoimmune polyendocrinopathy. *Lancet.* 1991;337(8750):1111–5. 10.1016/0140-6736(91)92784-Y 1674011

[82] de Jesus GR Agmon-Levin N Andrade CA: 14th International Congress on Antiphospholipid Antibodies Task Force report on obstetric antiphospholipid syndrome. *Autoimmun Rev.* 2014;13(8):795–813. 10.1016/j.autrev.2014.02.003 24650941

[83] Chen LM Zhang Q Si GX: Associations between thyroid autoantibody status and abnormal pregnancy outcomes in euthyroid women. *Endocrine.* 2015;48(3):924–8. 10.1007/s12020-014-0420-x 25209893

[84] Thangaratinam S Tan A Knox E: Association between thyroid autoantibodies and miscarriage and preterm birth: meta-analysis of evidence. *BMJ.* 2011;342:d2616. 10.1136/bmj.d2616 21558126 PMC3089879

[85] Iijima T Tada H Hidaka Y: Effects of autoantibodies on the course of pregnancy and fetal growth. *Obstet Gynecol.* 1997;90(3):364–9. 10.1016/S0029-7844(97)00283-4 9277645

[86] Mekinian A Cohen J Alijotas-Reig J: Unexplained Recurrent Miscarriage and Recurrent Implantation Failure: Is There a Place for Immunomodulation? *Am J Reprod Immunol.* 2016;76(1):8–28. 10.1111/aji.12493 26847715

[87] Piga M Serra A Deiana L: Brain perfusion abnormalities in patients with euthyroid autoimmune thyroiditis. *Eur J Nucl Med Mol Imaging.* 2004;31(12):1639–44. 10.1007/s00259-004-1625-7 15290119

[88] Montagna G Imperiali M Agazzi P: Hashimoto's encephalopathy: A rare proteiform disorder. *Autoimmun Rev.* 2016;15(5):466–76. 10.1016/j.autrev.2016.01.014 26849953

[89] Watt T Hegedü s L Bjorner JB: Is Thyroid Autoimmunity per se a Determinant of Quality of Life in Patients with Autoimmune Hypothyroidism? *Eur Thyroid J.* 2012;1(3):186–92. 10.1159/000342623 24783018 PMC3821477

[90] Bové KB Watt T Vogel A: Anxiety and depression are more prevalent in patients with graves' disease than in patients with nodular goitre. *Eur Thyroid J.* 2014;3(3):173–8. 10.1159/000365211 25538899 PMC4224229

[91] Avouac J Airò P Dieude P: Associated autoimmune diseases in systemic sclerosis define a subset of patients with milder disease: results from 2 large cohorts of European Caucasian patients. *J Rheumatol.* 2010;37(3):608–14. 10.3899/jrheum.090815 20110522

[92] Marinó M Ricciardi R Pinchera A: Mild clinical expression of myasthenia gravis associated with autoimmune thyroid diseases. *J Clin Endocrinol Metab.* 1997;82(2):438–43. 10.1210/jcem.82.2.3749 9024233

[93] Wiersinga WM Duntas L Fadeyev V: 2012 ETA Guidelines: The Use of L-T4 + L-T3 in the Treatment of Hypothyroidism. *Eur Thyroid J.* 2012;1(2):55–71. 10.1159/000339444 24782999 PMC3821467

[94] Coles AJ Wing M Smith S: Pulsed monoclonal antibody treatment and autoimmune thyroid disease in multiple sclerosis. *Lancet.* 1999;354(9191):1691–5. 10.1016/S0140-6736(99)02429-0 10568572

[95] Mandac JC Chaudhry S Sherman KE: The clinical and physiological spectrum of interferon-alpha induced thyroiditis: toward a new classification. *Hepatology.* 2006;43(4):661–72. 10.1002/hep.21146 16557537

[96] Antonelli A Ferri C Fallahi P: Hepatitis C: thyroid dysfunction in patients with hepatitis C on IFN-alpha therapy. *Nat Rev Gastroenterol Hepatol.* 2009;6(11):633–5. 10.1038/nrgastro.2009.168 19881515

[97] Stefan M Jacobson EM Huber AK: Novel variant of thyroglobulin promoter triggers thyroid autoimmunity through an epigenetic interferon alpha-modulated mechanism. *J Biol Chem.* 2011;286(36):31168–79. 10.1074/jbc.M111.247510 21757724 PMC3173071

[98] Spain L Diem S Larkin J: Management of toxicities of immune checkpoint inhibitors. *Cancer Treat Rev.* 2016;44:51–60. 10.1016/j.ctrv.2016.02.001 26874776

[99] Corsello SM Barnabei A Marchetti P: Endocrine side effects induced by immune checkpoint inhibitors. *J Clin Endocrinol Metab.* 2013;98(4):1361–75. 10.1210/jc.2012-4075 23471977

[100] Bliddal S Borresen SW Feldt-Rasmussen U: Thyroid Autoimmunity and Function after Treatment with Biological Antirheumatic Agents in Rheumatoid Arthritis. *Front Endocrinol (Lausanne).* 2017;8:179. 10.3389/fendo.2017.00179 28824542 PMC5534470

[101] Raterman HG Jamnitski A Lems WF: Improvement of thyroid function in hypothyroid patients with rheumatoid arthritis after 6 months of adalimumab treatment: a pilot study. *J Rheumatol.* 2011;38(2):247–51. 10.3899/jrheum.100488 21078720

[102] El Fassi D Nielsen CH Hasselbalch HC: Treatment-resistant severe, active Graves' ophthalmopathy successfully treated with B lymphocyte depletion. *Thyroid.* 2006;16(7):709–10. 10.1089/thy.2006.16.709 16889501

[103] Nielsen CH El Fassi D Hasselbalch HC: B-cell depletion with rituximab in the treatment of autoimmune diseases. Graves' ophthalmopathy the latest addition to an expanding family. *Expert Opin Biol Ther.* 2007;7(7):1061–78. 10.1517/14712598.7.7.1061 17665994

[104] Salvi M Vannucchi G Currò N: Efficacy of B-cell targeted therapy with rituximab in patients with active moderate to severe Graves' orbitopathy: a randomized controlled study. *J Clin Endocrinol Metab.* 2015;100(2):422–31. 10.1210/jc.2014-3014 25494967 PMC4318899

[105] Buch MH Smolen JS Betteridge N: Updated consensus statement on the use of rituximab in patients with rheumatoid arthritis. *Ann Rheum Dis.* 2011;70(6):909–20. 10.1136/ard.2010.144998 21378402 PMC3086093

[106] Hauser SL Waubant E Arnold DL: B-cell depletion with rituximab in relapsing-remitting multiple sclerosis. *N Engl J Med.* 2008;358(7):676–88. 10.1056/NEJMoa0706383 18272891

[107] Pescovitz MD Greenbaum CJ Krause-Steinrauf H: Rituximab, B-lymphocyte depletion, and preservation of beta-cell function. *N Engl J Med.* 2009;361(22):2143–52. 10.1056/NEJMoa0904452 19940299 PMC6410357

[108] Smith TJ Kahaly GJ Ezra DG: Teprotumumab for Thyroid-Associated Ophthalmopathy. *N Engl J Med.* 2017;376(18):1748–61. 10.1056/NEJMoa1614949 28467880 PMC5718164

[109] Baker KF Isaacs JD: Novel therapies for immune-mediated inflammatory diseases: What can we learn from their use in rheumatoid arthritis, spondyloarthritis, systemic lupus erythematosus, psoriasis, Crohn's disease and ulcerative colitis? *Ann Rheum Dis.* 2017; pii: annrheumdis-2017-211555. 10.1136/annrheumdis-2017-211555 28765121

